# The empirical support for the radical cure strategy for eliminating *Plasmodium vivax* in China

**DOI:** 10.1186/s12916-021-02214-y

**Published:** 2022-01-21

**Authors:** Guo-Jing Yang, Le-Yuan Shang, Xiao-Nong Zhou, Tamsin E. Lee, Bo Bi, Michael White, Thomas A. Smith, Melissa A. Penny

**Affiliations:** 1 Key Laboratory of Tropical Translational Medicine of Ministry of Education, the First Affiliated Hospital, Hainan Medical University, Haikou, Hainan 571199 People’s Republic of China; 2 Swiss Tropical and Public Health Institute, Socinstrasse, 57 4051 Basel, Switzerland; 3 University of Basel, Basel, Switzerland; 4 Henan Center for Disease Control and Prevention, Zhengzhou, Henan People’s Republic of China; 5 National Health Commission Key Laboratory on Biology of Parasite and Vector, National Institute of Parasitic Diseases, Chinese Center for Disease Control and Prevention, Shanghai, People’s Republic of China; 6 Institut Pasteur, Université de Paris, G5 Épidémiologie et Analyse des Maladies Infectieuses, Département de Santé Globale, F-75015 Paris, France

**Keywords:** Plasmodium vivax, Elimination, People’s Republic of China, Strategy, Model

## Abstract

**Background:**

With the recent certification by World Health Organization that the People’s Republic of China is malaria-free, it is timely to consider how elimination of malaria was completed in People’s Republic of China over the last 7 decades. Of the four widespread species of human malaria, *Plasmodium vivax* was the last to be eliminated by the national program of China. Understanding the incubation periods and relapses patterns of *P. vivax* through historical data from China is relevant for planning disease elimination in other malaria-endemic countries, with residual *P. vivax* malaria.

**Methods:**

We collated data from both published and unpublished malaria parasite inoculation experiments conducted between 1979 and 1988 with parasites from different regions of the People’s Republic of China. The studies had at least two years of follow-up. We categorized *P. vivax* incubation patterns via cluster analysis and investigated relapse studies by adapting a published within-host relapse model for *P. vivax* temperate phenotypes. Each model was fitted using the expectation-maximization (EM) algorithm initialized by hierarchical model-based agglomerative clustering.

**Results:**

*P. vivax* parasites from the seven studies of five southern and central provinces in the People’s Republic of China covering geographies ranging from the south temperate to north tropical zones. The parasites belonged to two distinct phenotypes: short- (10–19 days) or long-incubation (228–371 days). The larger the sporozoite inoculation, the more likely short incubation periods were observed, and with more subsequent relapses (Spearman’s rank correlation between the number of inoculated sporozoites and the number of relapses of 0.51, *p*-value = 0.0043). The median of the posterior distribution for the duration of the first relapse interval after primary infection was 168.5 days (2.5% quantile: 89.7; 97.5% quantile: 227.69 days). The predicted survival proportions from the within-host model fit well to the original relapse data. The within-host model also captures the hypnozoite activation rates and relapse frequencies, which consequently influences the transmission possibility of *P. vivax*.

**Conclusions:**

Through a within-host model, we demonstrate the importance of clearance of hypnozoites. A strategy of two rounds of radical hypnozoite clearance via mass drug administration (MDA) deployed during transmission (summer and autumn) and non-transmission (late spring) seasons had a pronounced effect on outbreaks during the malaria epidemics in China. This understanding can inform malaria control strategies in other endemic countries with similar settings.

**Supplementary Information:**

The online version contains supplementary material available at 10.1186/s12916-021-02214-y.

## Background

Although the global burden of *Plasmodium vivax* malaria dropped from 24.5 million cases in 2000 to 14.3 million cases in 2017 (a 42% reduction [1]), the control of *P. vivax* malaria remains neglected worldwide [2]. The reasons for this neglect include its lower prevalence and mortality compared with that of *P. falciparum* and the uneven distribution of *P. vivax* malaria in the world. Nearly 80–90% of *P. vivax* cases reported are from Asia, the Middle East, the Western Pacific, and the remainder occurring in Central and South America, and the poor understanding of the contribution of *P. vivax* to morbidity and mortality. The distinctive feature of *P. vivax* is relapse which can occur long after an initial attack [3], which is caused by dormant liver-stage parasites (hypnozoites). This creates more resilience in *P. vivax* transmission than that of *P. falciparum* in situations adverse to the transmission of the parasites. Hence, in the last mile, elimination of malaria in one country or region is frequently limited by residual *P. vivax.*

*P. vivax* was identified as a separate malaria species by Grassi and Feletti [4]. As far back as 1897, it was established that the incubation period of *P. vivax*, defined as the time between infection with the parasite by an *Anopheles* mosquito bite and the onset of the first clinical symptoms, could be much longer than that of *P. falciparum*. This incubation period was estimated to be 10 to 20 days [5]. Malaria therapy, whereby fever was induced in patients with neurosyphilis by infecting them with malaria parasites between the 1920s and the 1950s, gave a clearer understanding of relapse in *P. vivax* [6]. The *vivax* strains from Northern Europe and Russia often generated ineffective acute febrile illness, while strains from “Madagascar” successfully produced a short incubation infection with high fever [7]. Soldiers from the Korean war and patients from World War II who had received a large number of inoculations often experienced recurrences of malaria [8]. Korteweg recorded the mean monthly number of malaria cases in the village of Wormerveer, the Netherlands, from 1902 to 1923, and reported on long-latency *P. vivax* [9–11]. The demonstration that relapse is due to dormant hypnozoites in the liver dates back to 1948 by Shortt and Garnham [12]. The effects of recurrence of *P. vivax* via childhood and adult life are only rarely directly fatal. However, they can have major harmful consequences on individual growth and development, and on economic development at various levels, namely individual, family, community, and national [2].

In the 1940s, many strains were isolated from different geographical areas, and a systematic study was conducted on the length of the *P. vivax* incubation period and relapse patterns [3]. The effect of inoculum on the probability and pattern of relapse has been studied extensively in humans, in classic studies in chimpanzees [13], and in the Rhesus monkeys model with *P. cynomolg*i [14]. The World Health Organization (WHO) recommended classifying *P. vivax* into three types according to the length of incubation period and pattern of relapse. Type I (tropical strains) describes species with short incubation periods (10–20 days) and a series of relapses at short intervals (less than 6 months) after primary infection. Type II (Elizabeth strain) with two phenotypes describes infections of a short incubation period followed by a long dormant phase until a series of short interval relapses or a long-incubation period followed by a few relapses at short intervals after primary infection. Type III (temperate strain) describes types of long-incubation period (more than 6 months) with only a few short intervals of relapsing after primary infection [3].

In 1963, China reported the existence of long-incubation period *P. vivax* (types II or III) [15], and this was confirmed by human parasite inoculation experiments conducted between 1970s and 1980s [16]. Tracing back to the Second World War, long incubation *P. vivax* had already been observed among Japanese soldiers. In the 1980s, a large number of studies were conducted on the incubation period and relapse patterns, including both neonate cohort studies and human inoculation experiments [17–22]. From the late 1980s to the end of 1990s, there were limited reports or studies on malaria in China as malaria incidence was less than 1/10,000 in most areas of the country but from 2000 onward, malaria rebounded in the Huanghuai Plain, with small outbreaks and local epidemics in Anhui, Henan, and Jiangsu provinces [16, 23, 24]. New cases of malaria with no infection history in the previous year were often observed in spring, the non-transmission season. These cases could have played an essential role in the persistence of the infection.

Understanding the incubation periods and relapse patterns, and their geographical distributions, is critical to understanding how malaria control tools achieved their effects in the control stage before elimination, and to understanding the potential transmission risks in historical epidemic areas of *P. vivax* after malaria elimination in China. This epidemiological understanding is also relevant for predicting the risk of renewed transmission seeded by introductions. Exploring the types of incubation periods and relapses of *P. vivax* is of significance for the continuous improvement of malaria control strategies in the regions with similar settings. According to He Bin’s review, there were 71 counties (cities) in 16 provinces with both short- and long-incubation period *P. vivax* in China in 1986. These provinces cover a vast area between 29° 30′ and 39° 52′ north latitude, spanning from South Temperate Zone to North Tropical Zone. The proportion of *P. vivax* with short- versus long-incubation periods varied [25].

The purpose of the study is to (1) understand the *P. vivax* incubation and relapse patterns based on previous published and unpublished malaria parasite inoculation experiments conducted using volunteer participants in different regions of People’s Republic of China between 1979 and 1988 and (2) to use a within-host relapse model based on the *P. vivax* temperate phenotype found to correspond to the inoculation data, to explain the success of the radical cure strategy for malaria elimination adopted by China since the 1980s, especially in combating major epidemics.

## Methods

### *P. vivax* inoculation experiments

The data of two series of inoculation experiments conducted in provinces in China were collated, as follows:

A) In the Henan province: In 1979, Le-Yuan Shang and Shao-Wu Zhang, who engaged in malaria prevention and research in this province, led human *P. vivax* inoculation research in China.

B) In other provinces across China: following approaches used in the Henan province, many malaria professionals from other provinces, such as Guangxi, Hunan, Yunnan, and elsewhere, and their family members, volunteered to participate in *P. vivax* inoculation research in the 1980s [18, 20, 21, 26–29].

Ethical approval was not obligatory at the time and location of all these studies. All participants volunteered to join in the study. Most *P. vivax* inoculation studies followed standard procedures developed by Le-Yuan Shang, as follows:

#### Source of infections

Three to five milliliters of venous blood was withdrawn from a *P. vivax* patient and placed in a blood vessel covered by artificial placenta amniotic membrane to mimic human skin. Volunteers at each study location received inoculations from a single original donor.

#### Infecting and raising *Anopheles sinensis*

At the room temperature of 26 (± 1)°C, the vessel containing *P. vivax* infected blood was closely attached to the mosquito cage gauze. Newly emerged *An. sinensis* were fed on this blood and then kept at 26 (± 1)°C. Mosquitoes at different stages were randomly sampled and dissected to check for sporozoites in the salivary glands.

#### Participant enrollment criteria

Participants were volunteers including malaria control professionals and their family members from regions of low malaria endemicity. Inclusion criteria were voluntary participation, good health condition, no history of malaria infection within the past 3years, negative results for both blood smear and antibody tests (IFA) for malaria prior to commencing the experiment, and living in urban areas with relatively good protection from mosquito bites during transmission season (summer and autumn). While the possibility of re-infection was unlikely, it could not be excluded.

#### Inoculation experiment

Each volunteer was bitten by a single or multiple infected mosquito(es) until the mosquito(es) was (were) fully fed. Then, the blood-fed mosquitoes were dissected to confirm the existence of sporozoites in the salivary glands. Only when sporozoites were found were volunteers assumed to be inoculated successfully. The number of sporozoites was estimated using a high-fold magnification microscope. After applying light pressure to the sample slide’s cover glass, the number of sporozoites per field was counted. Slides with 400–00 or > 500 sporozoites per field of view were marked as 3+ or 4+, respectively. After applying heavy pressure to the cover glass, the number of sporozoites per field of view with 1–9, 10–49, 50–99, or > 100 sporozoites was marked as very few (vf), few (f), 1+, or 2+, respectively. Based on the number of mosquito bites and the total positively labeled (+) number or labeled (vf or f), the inoculum size was roughly estimated.

#### Observation of participants after inoculation

From 1 week after inoculation, daily thick blood smears were used to detect *P. vivax* parasites. If within a month there were no clinical episodes and blood tests remained negative, the frequency of blood exanimation was reduced to once per month. Whenever participant body temperature was higher than 37.5 °C, an immediate test for *P. vivax* was carried out. Patients with clinical episodes and parasite positive thick blood smears were administrated 1200 mg or 1500 mg chloroquine for 3 days to control clinical symptoms. After that, the relapse episodes of each participant were observed as described above. Each infected person was observed for two consecutive years without taking any anti-hypnozoite drugs. During this study period, infected volunteers were carefully protected from exposure to mosquito bites during the malaria transmission seasons (summer and autumn).

## Statistical analysis

### Investigation of incubation periods

Cluster analysis was applied to investigate the relationship between the number of inoculated sporozoites and the incubation period of primary infection by using the R package “mclust.” Model-based clustering was performed based on parameterized finite Gaussian mixture models. Each model was estimated using the expectation-maximization (EM) algorithm initialized by hierarchical model-based agglomerative clustering [30]. The package “mclust” selects the optimal model and the most appropriate number of clusters based on the values of the Bayesian information criterion (BIC) computed for 14 different types of Gaussians and a variety of model assumptions about the volume, shape, and orientation of clusters.

### Relapse pattern

Spearman correlation tests were conducted to evaluate the relationship between the number of inoculated sporozoites and number of relapses. Before doing correlation analysis, Shapiro-Wilk normality tests were carried out to evaluate the normality of each variable using the R function shapiro.test [31].

### Within-host relapse modeling to understand relapse

In order to further understand the relapse patterns, we calibrated a previous *P. vivax* within-host model to post inoculation data previously processed; White et al. [32] developed a series of *P. vivax* within-host models based on whether relapses track a tropical or temperate phenotype, capturing the hypnozoite activation rate and resulting relapse frequency which consequently influences the probability of transmission. In our follow-up investigation using this *P. vivax* within-host relapse modeling [6, 32], only temperate pattern data were analyzed.

The details of the model are as described by White et al. [32]. In the within-host model, a couple of assumptions are proposed. A censoring period of 14 days was assumed after the primary infection and relapse of *P. vivax* because if a further relapse occurred within 14 days of a previous relapse, it would have been undetected either because of (i) prophylaxis treatment of the initial relapse or (ii) new parasites emerging into the blood being masked by existing circulating blood stage parasites. Subjects were modeled as receiving *v* infectious bites, each producing an average of *N* hypnozoites. The modeled number of bites by a single mosquito consequently follows a geometric distribution of mean *N*, and the number of hypnozoites from *ν* infectious bites follows a negative binomial distribution with mean *νN* and failure parameter *ν*.

For each dataset, the Kaplan-Meier (KM) estimate point estimates, ${X}_j^t$, gives the probability that a participant would have experienced *j* relapses at time *t*, where *t* is the number of days since infection. ${X}_j^t\le j$ relapses at time *t*. Estimates were derived using the “survfit” function from the “survival” library in R. The 2.5% and 97.5% confidence bounds of the KM point estimate,${X}_{j,2.5\%}^t$ and ${X}_{j,97.5\%}^t$, were calculated for the proportion of participants who experienced no more than *j* relapses at a given time *t*.

The temperate phenotype within-host relapse model was used to stochastically simulate relapse times. The within-host model for relapse timings was calibrated to the data using approximate Bayesian computation (ABC) methods. We estimate five parameters, namely hypnozoite activation rate (α), the duration of long-latency (d_LL), number of hypnozoites (N), hypnozoite dispersion (r), and hypnozoite death rate (μ). This method was preferred over maximum likelihood methods due to the difficulty in constructing likelihoods that account for unobserved events during the censoring period.

We assumed gamma prior distributions for parameters N, r, 1/μ, and 1/α. For parameter d_LL, we assumed a uniform prior distribution (1–2000). The prior values are based on those in the original *P. vivax* temperate strain model of White et al. [32]. To assess the importance of the choice of prior distributions, we performed a sensitivity analysis where uniform prior distributions were assumed for all parameters (see Additional file 1: Fig. S3). We simulated 1,000,000 parameter sets, randomly sampled from the prior distributions. For each parameter, set the model prediction of ${Y}_j^t$ was stochastically simulated. The sampled parameter was accepted if and only if:

$$ {X}_{j,2.5\%}^t\le {Y}_j^t\le {X}_{j,97.5\%}^t\ \mathrm{for}\ \mathrm{all}\ j,\mathrm{and}\ t $$

The distribution of the five parameters accepted provides an approximation of the posterior distribution. Posterior medians and 95% credible intervals are provided.

## Results

### Infectious mosquito challenge studies

Based on the inoculation studies of *P. vivax* in Guangxi (GX), Guizhou (GZ), Henan Kaifeng (HN_KF), Henan Zhengzhou (HN_ZZ), Hunan, Yunnan Mengla (YN_ML), and Yunnan Weixi (YN_WX) provinces, the short incubation period of *P. vivax* in China was determined to be between 10 and 19 days (time to first clinical episode following parasite infection). The long-incubation period for primary infection was between 228 and 371 days (8.0–12.1 months).

**Fig. 1:**
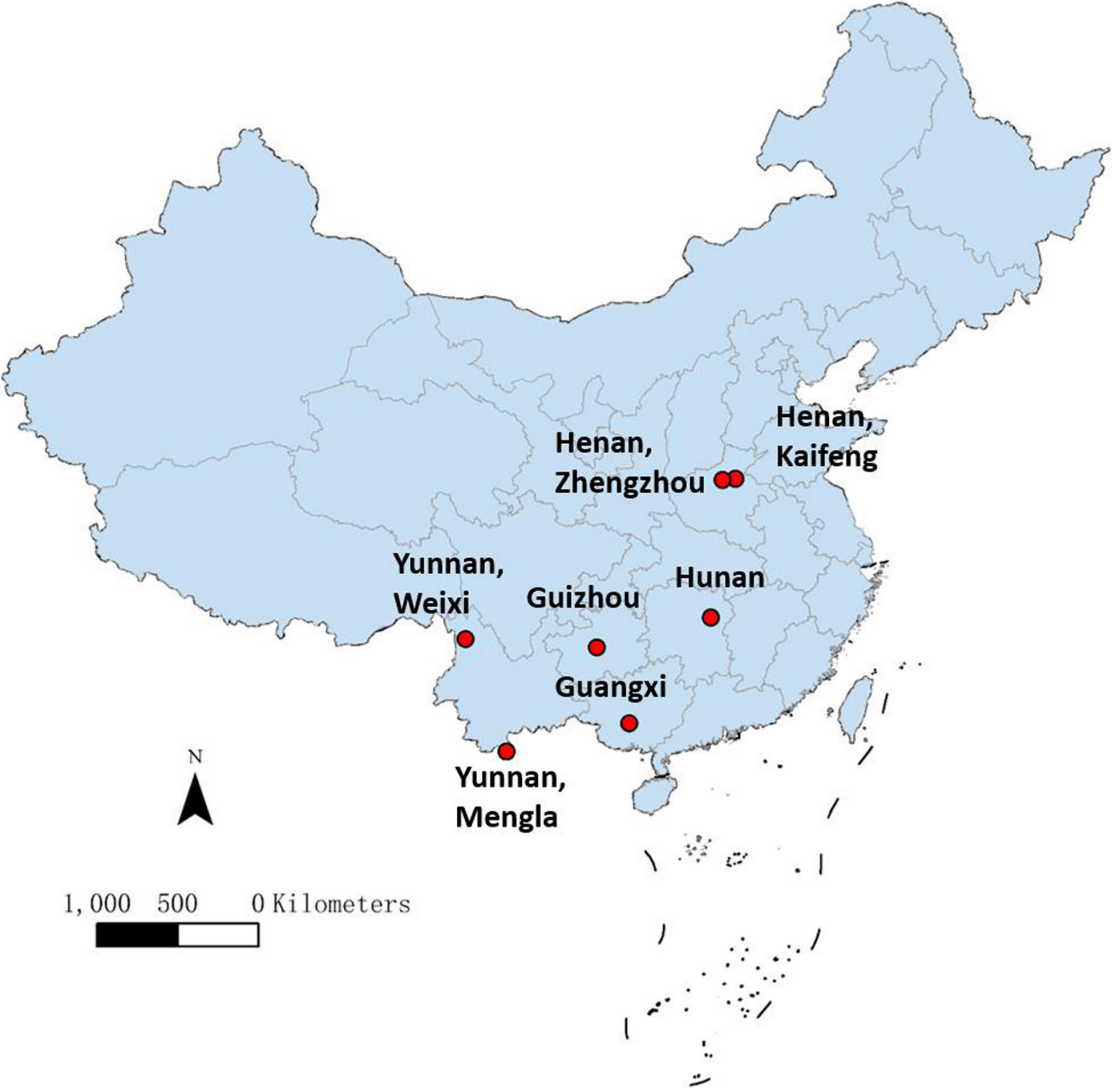
Locations of seven inoculation studies in five provinces in China

**Fig. 2:**
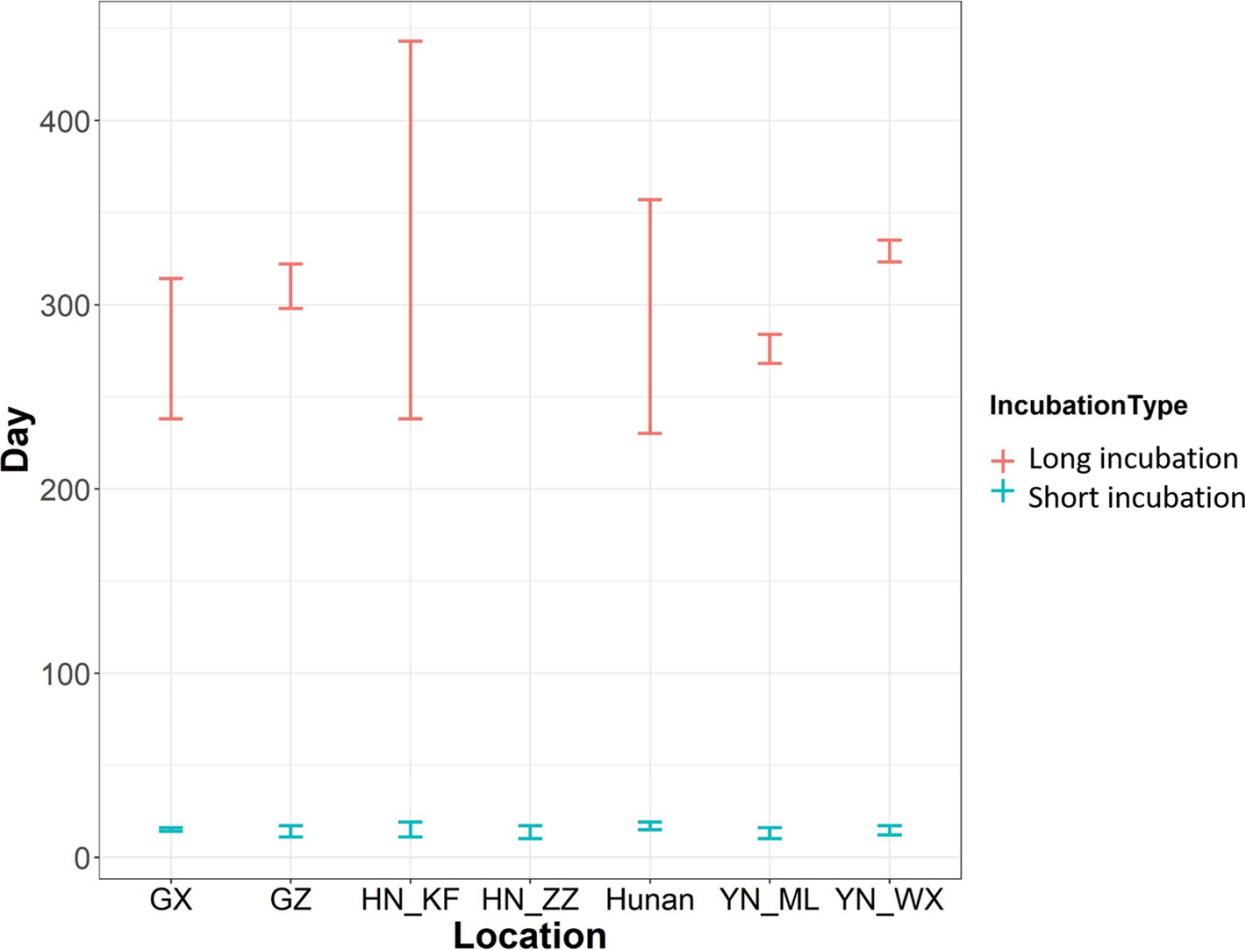
Minimum and maximum short/long incubation periods in seven inoculation studies. Guangxi (GX), Guizhou (GZ), Henan Kaifeng (HN_KF), Henan Zhengzhou (HN_ZZ), Hunan, Yunnan Mengla (YN_ML), and Yunnna_Weixi (YN_WX). There were no long incubation cases for study in Henan Zhengzhou (HN_ZZ)

Seven inoculation studies of *P. vivax* with high-quality data recorded in five provinces, namely Guangxi (GX), Guizhou (GZ), Henan Kaifeng (HN_KF), Henan Zhengzhou (HN_ZZ), Hunan, Yunnan ML (YN_ML), and Yunnan Weixi (YN_WX). The data are summarized in Figs. 1 and 2 and Table 1.

**Table 1:** Summary of incubation periods and relapse patterns of *P. vivax* inoculation experiments across different regions in China

Study location		GX	GZ	HN_KF	HN_ZZ	Hunan	YN_ML	YN_WX
Climatic zone (latitude)		South temperate zone (24° N)	South temperate zone (26° N)	South temperate zone (34° N)	South temperate zone (34° N)	South temperate zone (26° N)	North tropical zone (21° N)	South temperate zone (28° N)
No. participants		39(32 with episodes)	20(17 with episodes)	35 with episodes	19 with episodes	30 (27 with episodes)	39 (37 with episodes)	8 with episodes
Long incubation	**No. cases**	7	3	20	0	14	2	3
	**Time to first episode (days)**	238–314	298–322	268–371	No cases	230–357	268–284	228–335
	**1st relapse interval (days)**	41, 47, 50, 60, 109	46–48	42–68	No cases	51–80	0	No follow-up
	**Max relapse events**	1	1	1	No cases	1	0	No follow-up
Short incubation	**No. cases**	25	14	15	19	13	35	5
	**Time to first episode (days)**	14–16	11–17	11–19	11–19	15–19	10–16	13–17
	**1st relapse interval (days)**	252.5 ± 30.6	193–287	240–320	203–311	191–297	85%: long-term (228.8 ± 45.6) 15%: short-term (72 ± 20)	No follow-up
	**Max relapse events**	3	4	5	5	5	5	No follow-up

Guangxi (GX), Guizhou (GZ), Henan Kaifeng (HN_KF), Henan Zhengzhou (HN_ZZ), Hunan, Yunnan Mengla (YN_ML), and Yunnan Weixi (YN_WX)

### Direct sporozoite inoculation studies

The relapse pattern of all studies in temperate zones in China show high consistency (see Table 1). All cases with short incubation periods demonstrated a long-term interval until the first relapse. However, one study in the north tropical zone (Mengla, Yunnan) showed a more complex relapse form or so called intermediate-latency relapse. The first relapse of 85% of cases with short incubation periods were at long intervals (228.8 ± 45.6 days), which is the same as the temperate zone type, while the remaining 15% presented shorter intervals (72 ± 20 days), which is similar to the tropical type. The ratio of long and short-latency is about 6:1, which is almost the converse of findings in Aligarh, Uttar Pradesh, India with ratio of 1:4 [33].

Two studies with individual level data were used for cluster and correlation analysis (Table 2). One was led by B. Yang between 1983 and 1984 in Yunnan province. Among 39 infected volunteers, only 37 people (94.9%) developed clinical symptoms. After the first episode, 24 participants were only administered 1500 mg chloroquine for 3 days without taking primaquine. Another 15 participants were treated with chloroquine for 3 days and primaquine 210 mg for 14 days to clear hypnozoites at the time of initial symptoms. Another study was carried out in Guizhou province to observe the incubation period and relapsing pattern of *P. vivax* Guizhou strains [22].

**Table 2:** Number of inoculated sporozoite, incubation period, and time to relapse by volunteer

Volunteer	No. inoculated sporozoite (estimate of semi-quantified data)	No. of infective mosquito bites	Incubation period (day)	Time to relapse (days)				
				1st relapse	2nd relapse	3rd relapse	4th relapse	5th relapse
ID1	2600	10	11	166	255			
ID2	1100	5	11	242	285			
ID3	600	3	13	218				
ID4	300	1	13	197	244			
ID5	1400	10	11	66	137	210		
ID6	1100	7	10	54	134	187	240	297
ID7	400	5	12	217	277	369		
ID8	500	3	11	100	184	213	264	315
ID9	100	1	12	228	282	332		
ID10	100	1	12	208	278			
ID11	700	10	14	195	251			
ID12	900	7	12	160	202			
ID13	900	4	14	55	183	234		
ID14	900	3	14	185				
ID15	100	1	14	No relapse				
ID16	75	2	15	No relapse				
ID17	30	2	16	85	230	280		
ID18	10	2	284	No relapse				
ID19	25	1	268	No relapse				
ID20	1900	10	10	283	347			
ID21	1100	7	10	295	339			
ID22	700	5	14	299	341			
ID23	700	3	10	270				
ID24	25	1	10	269	332			
GD1^b^(7 participants)	2710^a^ (1000–8000, total 19000)	No data	13.8 (11–17)	247.7 (14 cases, 193–287)	314.6 (9 cases, 286–335)	398 ( 3 cases, 373,380,440)	459	
GD2^b^(7 participants)	278^a^ (150–800, total 1947)		14.1 (12–17)					
GD3_1	20	No data	298	No relapse				
GD3_2	23	No data	299	352				
GD3_3	42	No data	322	353				

The number of sporozoites in the salivary glands of each infected mosquitos was graded on a scale of 1+ to 26+. The total number of inoculated sporozoites was calculated and served as a rough indicator of the infective inoculum (the source document used did not contain details of these calculations)

^a^Averaged number of sporozoites among inoculation volunteers

^b^GD1 and GD2 are not individual-level data. We only had access to the average value of each group with 7 participants

#### Cluster and regression analysis

Among 14 proposed clustering models, the best fitting model was the VVI model (varying volume and shape, axis parallel orientation, diagonal covariance) with two clusters based on the BIC criterion (BIC = − 253.5) (see Additional file 1: Fig. S1).

**Fig. 3:**
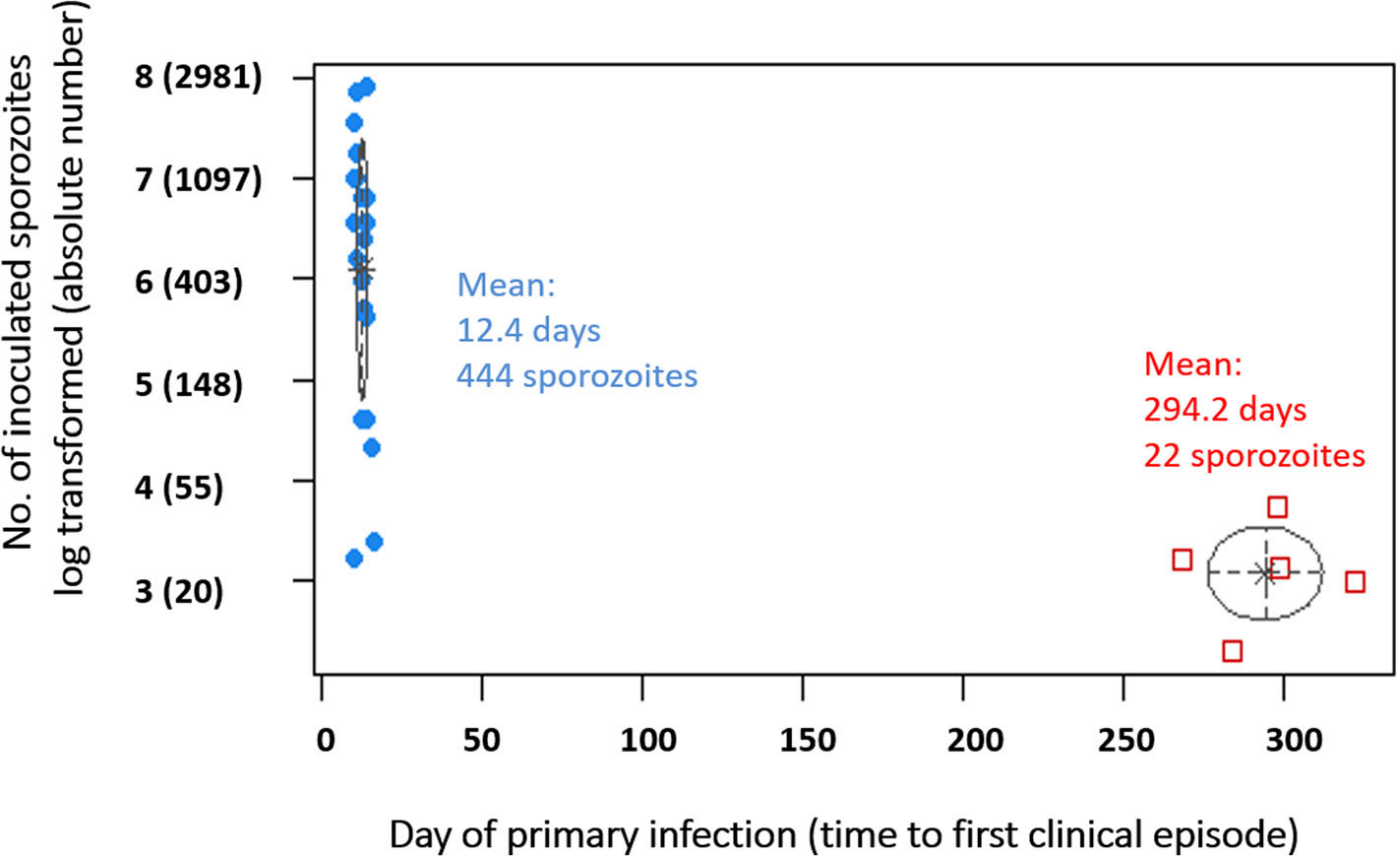
Cluster analysis of incubation period of inoculation studies. Blue dots indicate the first cluster with a mean incubation period of 12.4 days, while red squares denote the second cluster with a mean incubation period approaching 294.2 days

Our results indicate two clusters which are clearly separated in terms of the incubation periods (Fig. 3). The first cluster groups primary infections with a mean incubation period of 12.4 days with averaged 444 sporozoites inoculation (blue dots), while the second cluster groups infections with a mean incubation period approaching 294.2 days with averaged 22 sporozoites inoculation (red squares).

**Fig. 4:**
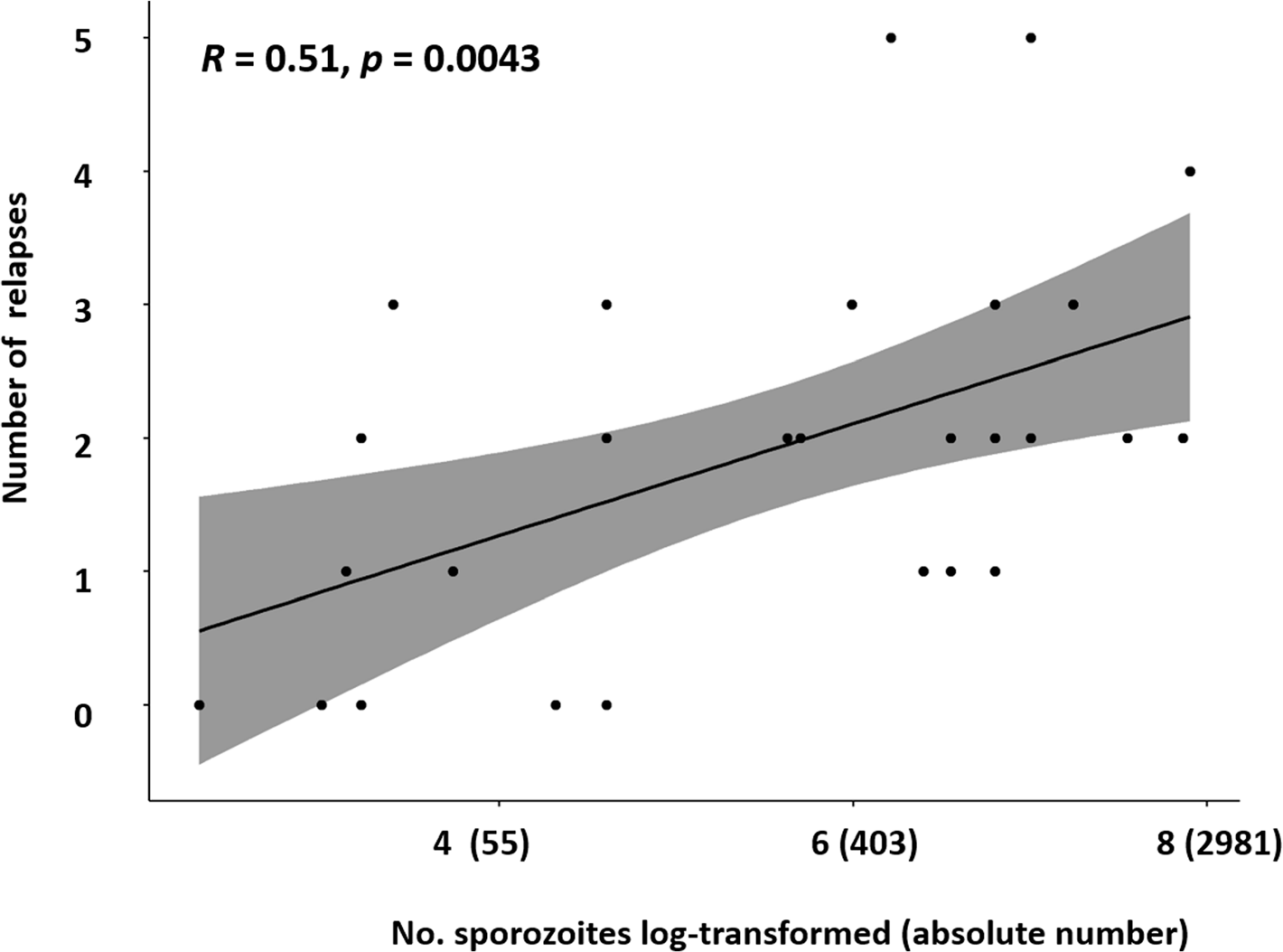
Spearman’s rank correlation between the number of inoculated sporozoites and relapses. The *x*-axis denotes number of sporozoites inoculated. The *y*-axis denotes the number of relapses. Shaded areas represent 95% confidence interval

The Spearman's rank correlation analysis indicates a statistically significant moderately positive correlation between the number of inoculated sporozoites and the number of relapses with value of 0.51 (*p*-value = 0.0043) (Fig. 4).

### Temperate within-host relapse model

**Fig. 5:**
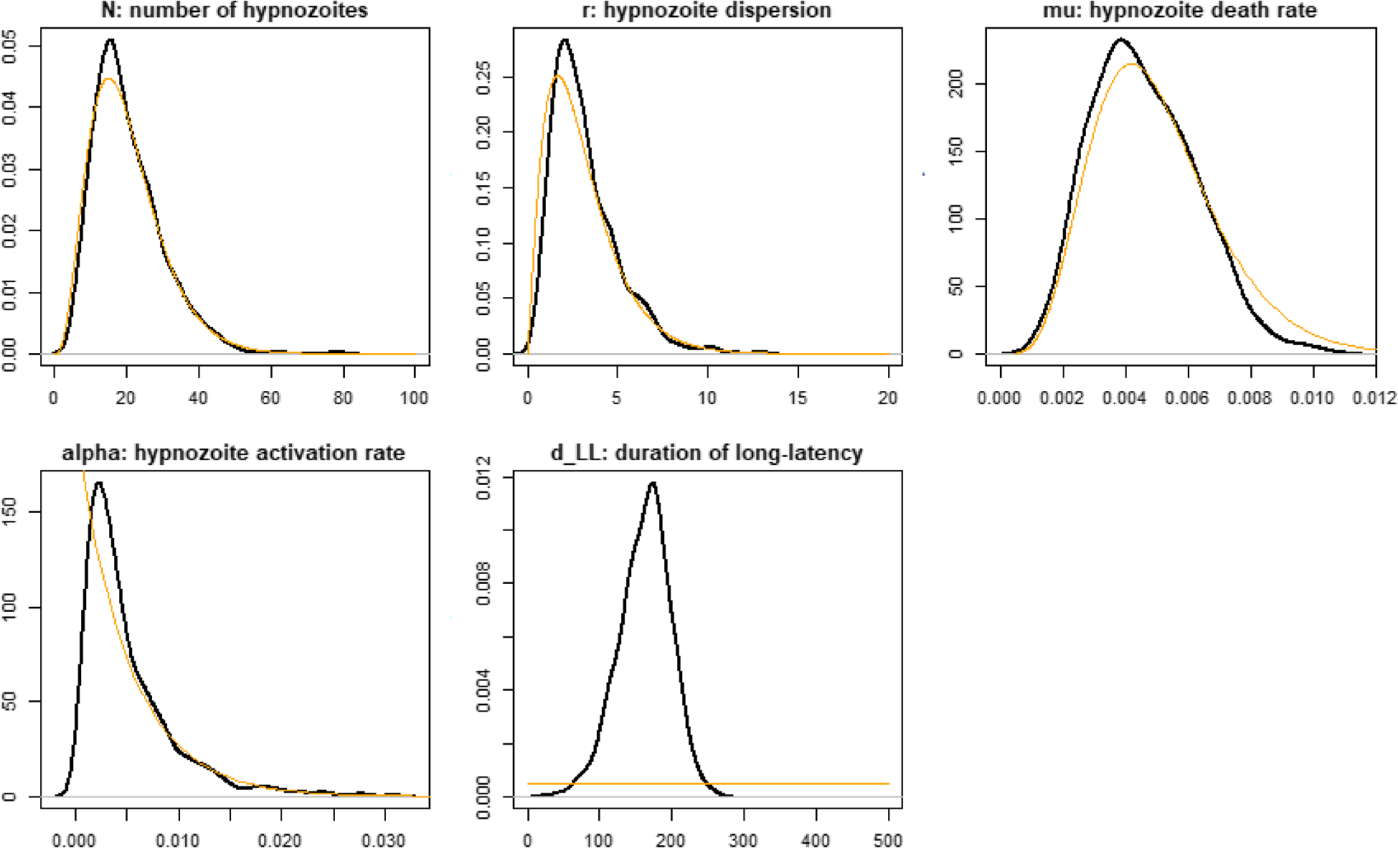
Posterior distribution of parameters of within-host model. The *x*-axis denotes each parameter’s value. The *y*-axis denotes the density of posterior distribution. The orange lines denote the prior distributions, based on the posterior distributions reported by White and colleagues [32] for the duration of long-latency (d_LL), a uniform prior distribution is assumed. The black lines denote the posterior distributions

**Fig. 6:**
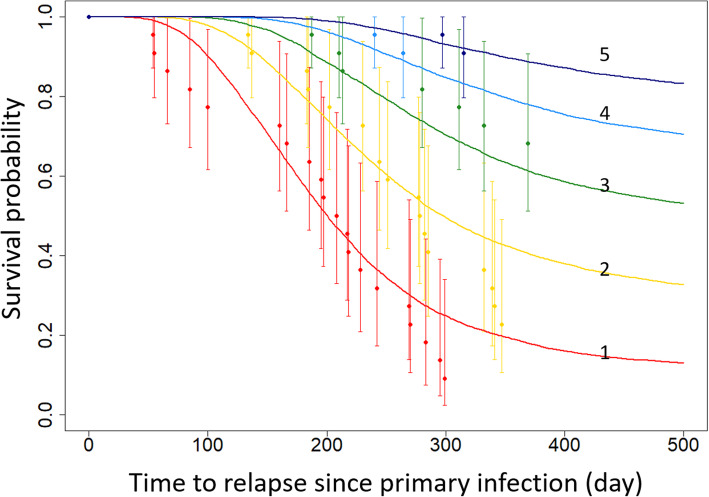
Within-host model prediction of the probability of survival for each relapse. The plot shows a comparison between model predicted times to relapse and the datasets described in Table 2. The curves represent the best-fit prediction for the within-host model. The five numbered curves represent five relapses. The dots indicate the survival probability of each relapse (based on raw data) with a 95% confidence interval (vertical bars). The survival analysis outcomes including 95% confidence intervals for the survival function at the time of each relapse are shown in the Additional file 1: Fig. S2

The posterior parameter estimates showed the 2.5% quantile, median, and 97.5% quantile of duration of long latency were 89.70, 168.5, and 227.69 days, respectively (Table 3, Fig. 5). Within-host model predicted survival proportions fit original relapse data well (Fig. 5, Additional file 1: Fig. S2). The within-host model captured the hypnozoite activation rates and relapse frequencies, which consequently influences the transmission possibility of *P. vivax* (Fig. 6).

**Table 3:** Posterior parameter estimated for within-host model fitted to data on multiple relapses

Parameter	Median	2.5% quantile	97.5% quantile
**Number of hypnozoites**	18.49	7.38	43.89
**Hypnozoite dispersion**	2.82	0.82	8.43
**Hypnozoite death rate**	0.0044	0.0018	0.0083
**Hypnozoite activation rate**	0.0041	0.00078	0.018
**Duration of first relapse interval after primary infection**	168.58	89.70	227.69

## Discussion

In China, both short- and long-incubation period phenotypes of *P. vivax* were observed in various studies, covering geographies ranging from the south temperate to north tropical zones [25]. We found, in China, the short- and long-incubation periods of *P. vivax* were 10–19 days and 228–443 days (8.0–14.7 months), respectively. The malaria transmission season in central China generally runs from early June to early November. However, before China’s malaria control and elimination program was launched, a large number of malaria cases were observed during the non-transmission season (from February to April), which could be well explained by the first relapse episode of long-incubation cases. Previously, Yang and colleagues performed a descriptive analysis using historical monthly incidence data from the Henan province (central China) and observed clear double peaks of malaria episodes during the 1960s and 1970s further supporting our findings for both short- and long-period incubations [34].

Our incubation period estimates are similar to those found by Nishiura et al. [35] from 416 people living in Korea between 2000 and 2003, with a mean long-incubation period of 48.2 weeks (95% CI: 46.8–49.5), equivalent to 337 days [35] (demonstrated by the maximum likelihood method used to fit the frequency distribution of long- and short-incubation periods throughout the year). Swellengrebel et al. using data from the Netherlands also demonstrated two peaks of malaria cases representing delayed primary illness in late spring and early primary illness plus relapse in summer and autumn [10, 36]. Similar findings were also observed by Zhou Wentao [37], namely, that the distribution of cases with long- and short-incubation periods formed peaks in summer and autumn, as well as in the late spring of the following year, with an average 9.5 months (285 days) [37] in Changde City, Hunan Province of China using 7 years of data. Using cluster analysis, we also found that the long-incubation period is around 294 days after sporozoite inoculation.

If the period between infection and the last relapse plus 2 weeks self-healing course [18] is regarded as the natural life span of *P. vivax* in humans, then the longest *P. vivax* infection period based on various studies and observations in China is between 342 and 447 (geometric mean 366.6 + 14) days. This length is far less than previously believed at 3 years or longer in untreated *P. vivax* [19]. Consistent with the results here, in a South Korean study, a total of 79 eligible cases were selected to estimate the length of the long-incubation period, concluding that it was 337 days (95% CI: 328–347) [35]. Therefore, three successive years without indigenous malaria cases reported suffices to allow certification of malaria elimination. This can be applied to China, which postponed certification due to the COVID-19 pandemic and announced malaria elimination on 30 June 2021.

A plausible explanation for the difference between short- and long-incubation is that these patterns are a result of two populations of sporozoites of *P. vivax* within each inoculation. Short-incubation-sporozoites (SISs), called *Tachyspororozoites* [38], develop and proliferate rapidly, invade red blood cells, and then develop into exoerythrocytic schizonts. Long-incubation-sporozoites (LISs), called *Bradysporozoites* [38], do not immediately develop and proliferate but initially remain dormant in the liver and develop into hypnozoites. After varying periods of dormancy, hypnozoites develop and proliferate in batches and enter red blood cells causing multiple relapses [39]. In other words, long incubation infections could be considered as asymptomatic sub-patent infections who eventually became patent. In temperate settings, LISs are present in great excess with a much smaller proportion of sporozoites characterized by SISs [40]. Garnham [3] already recognized Korean *P. vivax* strains as a limiting case of almost exclusively LISs.

The assumption of two kinds of sporozoites can explain the conclusion that the duration of incubation, or the inter relapse interval(s) (for relapses with genetically homologous parasites), was determined by inoculum size [41]. When the number of inoculated sporozoites is small, there is an increased likelihood that there will not be a malarial episode within the short incubation period. Primary illness occurs by nine or 10 months, or even longer, which our research supports. The more sporozoites which are inoculated, the more likely there will be short incubation periods and with a higher number of subsequent relapses. Experiments with quantitative inoculation of sporozoites by B. Yang in the Hunan province of China also confirmed this finding [19, 42]. Different numbers of sporozoites were immediately injected into 21 volunteers by endothelial method after vitro extraction. The 16 volunteers inoculated with more than 10,000 sporozoites all showed short incubation periods of 15 ± .4.5 days. Five volunteers with 100 sporozoites all had long incubation periods, 312 ± 40.7 days. Ten volunteers with 1000 sporozoites inoculation presented both short (14.4 ± 0.8 days) and long (282 ± 29.9 days) periods. This study indicated that the ratio between SISs and LISs was around 1:1000, which is similar to the findings of Garnham and colleagues applying the North Korean strain in a chimpanzee model [3].

Long-short-long-incubation pattern rotation has been observed in many other studies [25, 43]. Volunteers infected by one sporozoite-positive mosquito, or a small number of sporozoites, developed a long-incubation period phenotype. A batch of newly emerged *An. sinensis* was then infected using these volunteers’ blood. Another group of volunteers were then infected with multiple sporozoite-positive mosquitoes or a large quantity of sporozoites from this batch. These volunteers had short incubation period phenotypes. The long- incubation period phenotype recurred when the previous steps in the experiment were repeated, and new healthy volunteers were bitten by one sporozoite-positive mosquito or inoculated with a small amount of sporozoites. If multiple sporozoites lead to relapses in the same human host, then the relapse pattern is classified by the period corresponding to the first one to relapse. The separation into two classes (Fig. 3) is a question of whether at least one *Tachyspororozoite* happens to survive. A rotation study with a long-short-long-incubation pattern, which used the same malaria parasite strain as we did for our study, reenforces the theory that the short- or long-incubation phenotypes are determined by the amount of inoculated sporozoites instead of by the region where the parasite strain originated [17, 25]. Using climatic zone as an indicator to classify malaria types may not be a proper way to make classifications, which was pointed out by Coatney in 1971. Coexistence of short- and long-relapse phenotypes of *P. vivax* have been demonstrated through genotyping strains from Northeast India and Nepal [44, 45]. However, in view of the clear differences in duration of incubation and frequency of relapse, it is worth developing effective anti-malaria strategies according to phenotypes instead of by climatic zone.

Although the sporozoite inoculation experiments attempted to mimic natural infections, many parameters could not be considered, for instance, immunity. The acquisition of blood stage immunity may also affect the length of incubation or the relapse period [46]. In the case of malaria outbreaks in the non-immune or low-immunity populations, patients with fever episodes between days 3 and 6 showed high infectivity to mosquitoes—an infection rate of between 72 and 100%. The number of oocysts per positive mosquito varied from 27 to 316. With the increase in blood stage immunity, the mosquito infection rate drops sharply, especially if the number of oocysts within mosquitoes was reduced to 1–7, and this status lasted until transmission ended [46]. It is therefore inferred that the ratio of the two phenotypes of incubation periods in the epidemic area depends also on the level of immunity in the population during the epidemic. In the early- and mid-stages of a malaria outbreak, due to the high infectious rate and infection intensity, a large number of sporozoites could be inoculated into a human host via mosquito bites, which would lead to a large number of short latency cases. With the increase in immunity among the general population in the late stage of a malaria endemic, infectivity of mosquitoes significantly declined. Long-latency malaria cases would be the dominant type owing to the small number of sporozoites inoculated, which is consistent with our findings. In our within-host model, although the quantitative relationship between the number of relapses and the amount of sporozoites is considered, the immunity effect was not considered and should be a focus in future studies. Furthermore, we lack evidence for correlation between the inoculum size and the sporozoite load in the salivary glands. Further investigation of this is warranted using both experimental approaches and within-host modeling.

Both the outcomes from the volunteer study and model simulation results provided evidence of the coexistence of short- and long-latency *P. vivax* phenotypes with subsequent relapse, which was driven by inoculum size. As early as the 1960s, malaria control experts proposed a strategy to implement two rounds of radical cure mass drug administration (MDA). Through observation and field experiences, the strategies were specifically two rounds of radical hypnozoite clearance MDA deployed during transmission (summer and autumn) and non-transmission (late spring) seasons. Implementation of this strategy had a pronounced effect on controlling outbreaks during epidemics in the 1960s and 1970s. This strategy likely cleared both asymptomatic carriers and long-incubation patients, who would otherwise serve as the main contributors in the next transmission season. The treatment regimens were changing over time, beginning with pyrimethamine (100 mg) combining 5- or 8-day primaquine (150 or 180 mg, respectively) in the 1960s and early 1970s to a 3-day-chloroquine (1200 mg) combining 8-day primaquine (180 mg) administration in the late 1970s and onwards. In some regions in China, treatment was strengthened with pyrimethamine salt (5 mg per day) administered between mid-June and mid-September. However, large-scale MDA was not implemented when the incidence of malaria was below 5%. He Bin and colleagues proposed a substitute strategy to a village when incidence rates were less than 3%, namely only positive episodes receive malaria chemotherapy and during non-transmission seasons a 4-day chloroquine and primaquine treatment could be given. Further strategies were proposed for regions where *An. sinensis* was the only vector, and when prevalence was less than 1%, this involved only targeting hypnozoite treatment to symptomatic patients as a substitute for the “two round radical cure MDA strategy.” This modified strategy was applied in two pilot areas in Huanghuai region in China and achieved satisfactory social and economic benefits [24]. In China’s malaria elimination stage since 2010, the “1-3-7” surveillance response system together with a targeted high precision radical cure led to the achievement of malaria elimination [47].

## Conclusions

Through a within-host model, we demonstrate the importance of clearance of hypnozoites. A strategy of two rounds of radical hypnozoite clearance via mass drug administration (MDA) deployed during transmission (summer and autumn) and non-transmission (late spring) seasons had a pronounced effect on outbreaks during the malaria epidemics in China. This understanding can inform malaria control strategies in other endemic countries with similar settings.

## Supplementary Information

**Additional file 1: Fig. S1.** Bayesian Information Criterion (BIC) for different models of clustering. The x-axis denotes number of components (clusters). The y-axis denotes the BIC value. A number of 14 different curves representing various types of Gaussians and a variety of model assumptions about the volume, shape, and orientation of clusters. **Fig. S2**. The survival function at the time of each relapse. Survival analysis outcomes including 95% confidence interval: survival function at the time of each relapse. The horizontal axis (x-axis) represents time in days, and the vertical axis (y-axis) shows the probability for each relapse. The lines represent survival curves derived from original data. The dash lines denote the 95% confidence intervals. **Fig. S3**.. Posterior distribution of parameters of the within-host model with uniform prior information. The x-axis denotes each parameter’s value. The y-axis denotes the density of posterior distribution. The orange lines denote the prior distributions, based on the posterior distributions reported by White and colleagues [32] for the duration of long-latency (d_LL), and parameters (N, r, mu, alpha) a uniform prior distribution is assumed. The black lines denote the posterior distributions.

## Data Availability

All data used in this study have been published in the manuscript.

## Abbreviations

ABC: Approximate Bayesian computation
BIC: Bayesian information criterion
EM: Expectation-maximization
IFA: Blood smear and antibody tests
KM: Kaplan-Meier
LISs: Long-incubation-sporozoites
MDA: Mass drug administration
SISs: Short-incubation-sporozoites
VVI: Varying volume and shape, axis parallel orientation, diagonal covariance model
