# Supplementary material for: The empirical support for the radical cure strategy for eliminating Plasmodium vivax in China
Source: BMC Med. 2022 Jan 21;20:17. doi: 10.1186/s12916-021-02214-y (PMC8776510; doi:10.1186/s12916-021-02214-y)

**Additional file1:**

**Fig. S1 Bayesian Information Criterion (BIC) for different models of clustering.** The x-axis denotes number of components (clusters). The y-axis denotes the BIC value. A number of 14 different curves representing various types of Gaussians and a variety of model assumptions about the volume, shape, and orientation of clusters.


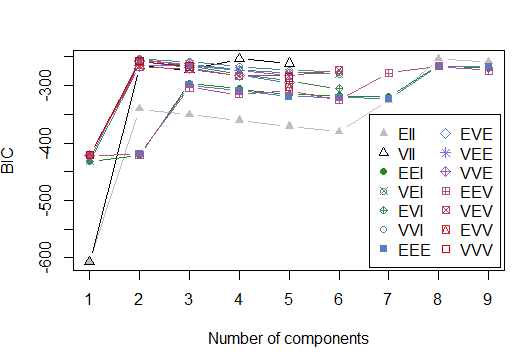


**Notes:** Abbreviation of 14 types of Gaussians and model assumptions

"EII" equal volume and round shape, spherical covariance

"VII" varying volume, round shape, spherical covariance

"EEI" equal volume and shape, axis parallel orientation diagonal covariance

"VEI" varying volume, equal shape, axis parallel orientation, diagonal covariance

"EVI" equal volume, varying shape, axis parallel orientation, diagonal covariance

"VVI" varying volume and shape, axis parallel orientation, diagonal covariance

"EEE" equal volume, shape and orientation, ellipsoidal covariance

"EVE" equal volume, varying shape, equal orientation, ellipsoidal covariance

"VEE" varying volume, equal shape and orientation, ellipsoidal covariance

"VVE" varying volume and shape, equal orientation, ellipsoidal covariance

"EEV" equal volume and equal shape, varying orientation, ellipsoidal covariance

"VEV" varying volume, equal shape, varying orientation, ellipsoidal covariance

"EVV" equal volume, varying shape, varying orientation, ellipsoidal covariance

"VVV" varying volume, shape and orientation, ellipsoidal covariance


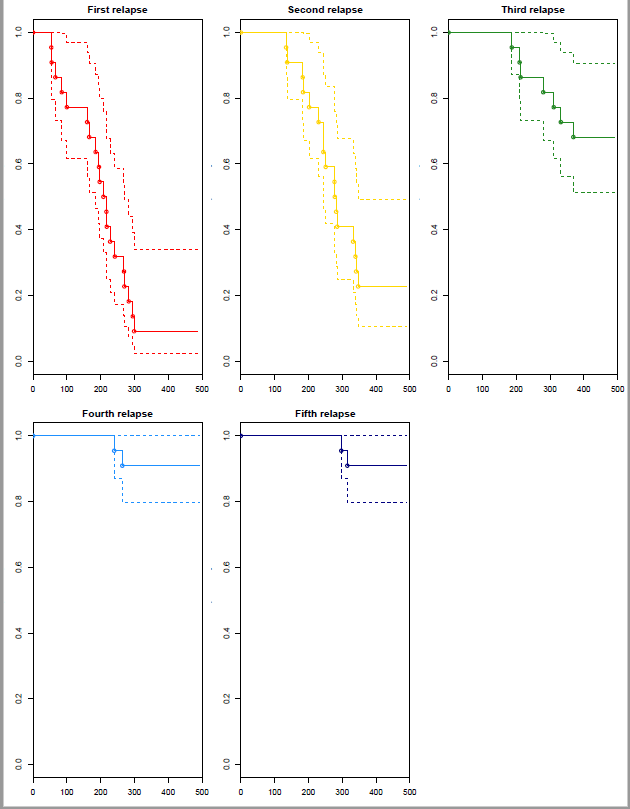
**Fig. S2 The survival function at the time of each relapse.** Survival analysis outcomes including 95% confidence interval: survival function at the time of each relapse. The horizontal axis (x-axis) represents time in days, and the vertical axis (y-axis) shows the probability for each relapse. The lines represent survival curves derived from original data. The dash lines denote the 95% confidence intervals.

**Fig. S3. Posterior distribution of parameters of the within-host model with uniform prior information.** The x-axis denotes each parameter’s value. The y-axis denotes the density of posterior distribution. The orange lines denote the prior distributions, based on the posterior distributions reported by White and colleagues(32) for the duration of long-latency (d_LL), and parameters (N, r, mu, alpha) a uniform prior distribution is assumed. The black lines denote the posterior distributions.


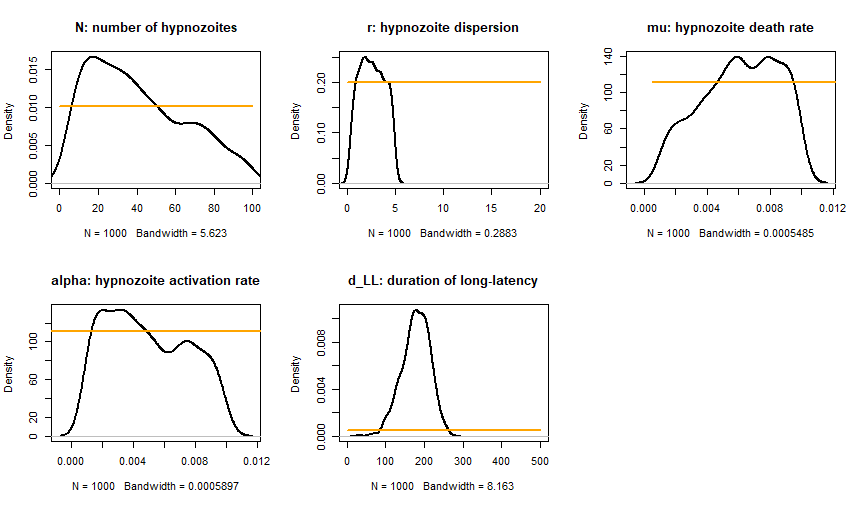

Supplement: Supplementary file 1 — Additional file 1: Fig. S1. Bayesian Information Criterion (BIC) for different models of clustering. The x-axis denotes number of components (clusters). The y-axis denotes the BIC value. A number of 14 different curves representing various types of Gaussians and a variety of model assumptions about the volume, shape, and orientation of clusters. Fig. S2. The survival function at the time of each relapse. Survival analysis outcomes including 95% confidence interval: survival function at the time of each relapse. The horizontal axis (x-axis) represents time in days, and the vertical axis (y-axis) shows the probability for each relapse. The lines represent survival curves derived from original data. The dash lines denote the 95% confidence intervals. Fig. S3.. Posterior distribution of parameters of the within-host model with uniform prior information. The x-axis denotes each parameter’s value. The y-axis denotes the density of posterior distribution. The orange lines denote the prior distributions, based on the posterior distributions reported by White and colleagues [32] for the duration of long-latency (d_LL), and parameters (N, r, mu, alpha) a uniform prior distribution is assumed. The black lines denote the posterior distributions. [file 12916_2021_2214_MOESM1_ESM.docx]
